# Improvement in coronary microvascular dysfunction after transcatheter aortic valve implantation leading to positive fractional flow reserve and percutaneous coronary intervention: a case report

**DOI:** 10.1093/ehjcr/ytaf649

**Published:** 2025-12-13

**Authors:** Kosuke Fujita, Kyohei Onishi, Ayano Yoshida, Hiroki Matsuzoe, Gaku Nakazawa

**Affiliations:** Division of Cardiology, Department of Medicine, Kindai University Faculty of Medicine, 1-14-1 Mihara-dai, Minami-ku, Sakai, Osaka 590-0197, Japan; Division of Cardiology, Department of Medicine, Kindai University Faculty of Medicine, 1-14-1 Mihara-dai, Minami-ku, Sakai, Osaka 590-0197, Japan; Division of Cardiology, Department of Medicine, Kindai University Faculty of Medicine, 1-14-1 Mihara-dai, Minami-ku, Sakai, Osaka 590-0197, Japan; Division of Cardiology, Department of Medicine, Kindai University Faculty of Medicine, 1-14-1 Mihara-dai, Minami-ku, Sakai, Osaka 590-0197, Japan; Division of Cardiology, Department of Medicine, Kindai University Faculty of Medicine, 1-14-1 Mihara-dai, Minami-ku, Sakai, Osaka 590-0197, Japan

**Keywords:** Aortic stenosis, Transcatheter aortic valve implantation, Fractional flow reserve, Index of microcirculatory resistance, Coronary microvascular dysfunction, Case report

## Abstract

**Background:**

The management of coronary artery disease in patients with severe aortic stenosis is controversial, with no consensus on optimal revascularization strategies. The validity of using fractional flow reserve to assess ischaemia in this population is debated. Conflicting results have arisen regarding the impact of transcatheter aortic valve implantation on fractional flow reserve values. We present the case of a patient with severe aortic stenosis and intermediate left anterior descending artery stenosis, in whom the fractional flow reserve and the index of microcirculatory resistance suggested the presence of coronary microvascular dysfunction prior to transcatheter aortic valve implantation. However, after valve replacement, the fractional flow reserve and the index of microcirculatory resistance indicated physiologically significant ischaemia, prompting intervention.

**Case summary:**

An 82-year-old woman presented with paradoxical low-flow, low-gradient severe aortic stenosis and intermediate left anterior descending artery stenosis. The fractional flow reserve was borderline, and the index of microcirculatory resistance was elevated before transcatheter aortic valve implantation, indicating the presence of coronary microvascular dysfunction. Six months after valve replacement, the fractional flow reserve declined to 0.64 with an improved index of microcirculatory resistance, prompting a successful percutaneous coronary intervention. Subsequent cardiac magnetic resonance imaging revealed reverse remodelling with a reduced left ventricular mass.

**Discussion:**

This case illustrates that transcatheter aortic valve implantation may unmask coronary ischaemia by restoring microvascular vasodilatory capacity in patients with coronary microvascular dysfunction. It also highlights the importance of reassessing coronary physiology in selected patients following transcatheter aortic valve implantation.

Learning points

- Severe aortic stenosis increases left ventricular afterload, resulting in hypertrophy and impaired coronary microvascular vasodilation, which may lead to underestimation of ischaemia by fractional flow reserve.
- Transcatheter aortic valve implantation relieves afterload and decreases myocardial oxygen demand, facilitating reverse remodelling and recovery of coronary microvascular function.
- Reassessment of coronary physiology after transcatheter aortic valve implantation may provide important information for optimal management in selected patients.

## Introduction

The optimal management of coronary artery disease (CAD) in patients with aortic stenosis (AS) is controversial. The ACTIVATION and NOTION-3 trials discussed revascularization strategies for patients with severe AS; however, no consensus was reached.^1,2^ In particular, the validity of using fractional flow reserve (FFR) to assess ischaemia in this population is debated. Conflicting results have been obtained regarding the impact of transcatheter aortic valve implantation (TAVI) on FFR values.^3–5^ Stoller *et al*.^3^ demonstrated an increase in hyperaemic flow and a decrease in FFR immediately after TAVI, suggesting improved coronary vasodilation capacity due to afterload reduction.

In contrast, Stundl *et al*.^4^ observed that FFR remained stable post-TAVI in most cases, indicating minimal influence of valve replacement on pressure-derived ischaemia assessment. Adding to this complexity, Zelis *et al*.^5^ discussed potential mechanisms by which FFR may paradoxically decrease after TAVI, including increased myocardial oxygen demand and changes in coronary microvascular resistance. We present the case of a patient with severe AS with intermediate left anterior descending artery (LAD) stenosis and borderline FFR before TAVI, accompanied by an elevated index of microcirculatory resistance (IMR). At the 6-month follow-up after TAVI, the FFR had significantly declined, while the IMR had improved, leading to the decision to perform percutaneous coronary intervention (PCI).

## Summary figure

**Figure:**
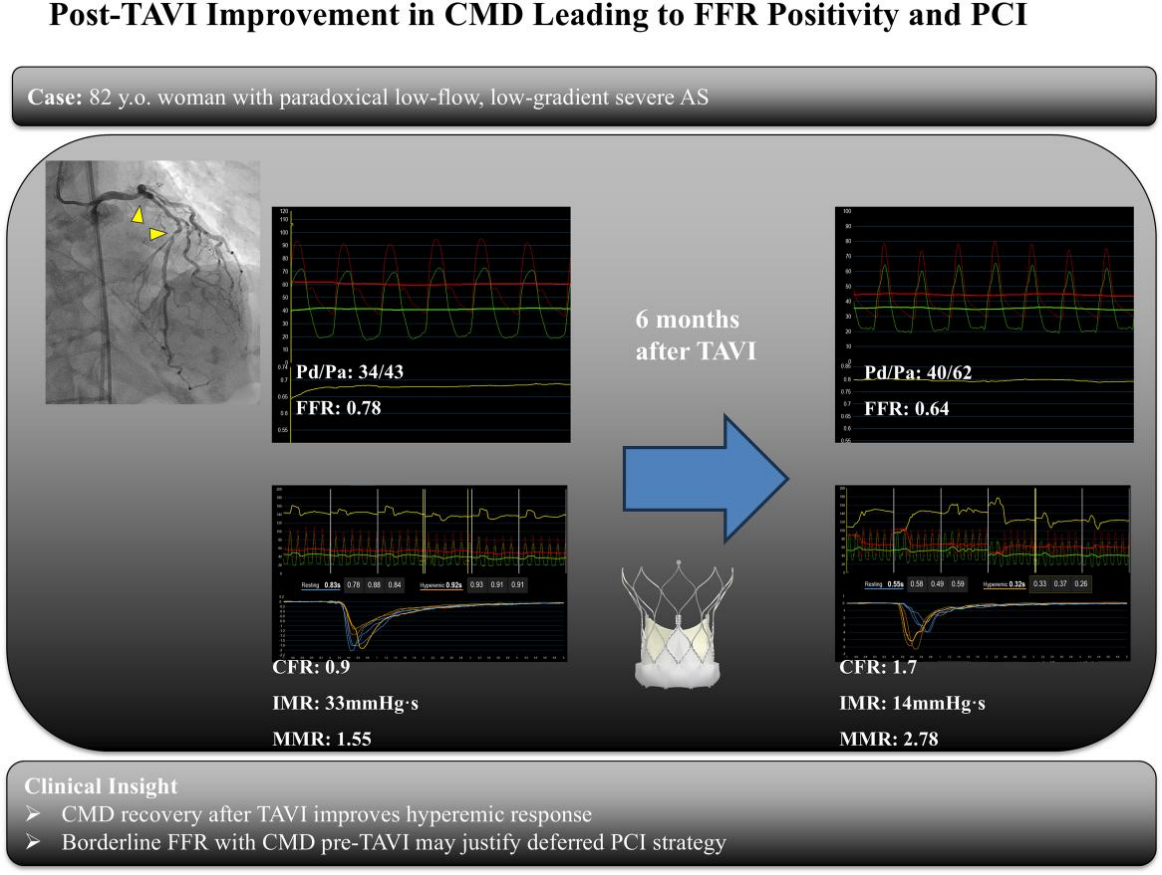


## Case presentation

### Pre-procedural evaluation

An 82-year-old woman with moderate AS, diagnosed 3 years earlier, had been followed conservatively at another hospital. She underwent emergency surgery for a strangulated ileus, requiring resection of 130 cm of necrotic intestine. Following surgery, she developed heart failure and was referred to our institution for further evaluation and consideration of advanced therapeutic interventions. Her clinical frailty scale score was 4, and her body surface area was 1.34 m². She presented with New York Heart Association (NYHA) class III dyspnoea upon exertion.

### Echocardiographic findings

Transthoracic echocardiography (TTE) before TAVI showed preserved left ventricular systolic function (left ventricular ejection fraction 66%) without apparent wall motion abnormalities, with a stroke volume index of 42 mL/m², a transaortic valve peak velocity of 3.5 m/s, a mean pressure gradient of 30 mmHg, and an aortic valve area of 0.68 cm² (by continuity equation). These findings were consistent with severe paradoxical low-flow, low-gradient (LFLG) AS. Other detailed echocardiographic parameters are summarized in Supplementary material online, *Table S1*.

### Heart team decision and computed tomography evaluation

After comprehensive discussion, our institutional heart team determined that TAVI was the most appropriate therapeutic strategy for severe symptomatic paradoxical LFLG AS. Pre-procedural contrast-enhanced computed tomography showed heavy calcification of all three cusps, with minimal calcification at the annulus and sinotubular junction. The total aortic valve Agatston calcium score was 2 150, indicating severe leaflet calcification.

### Coronary angiography and physiological assessment

**Figure 1:**
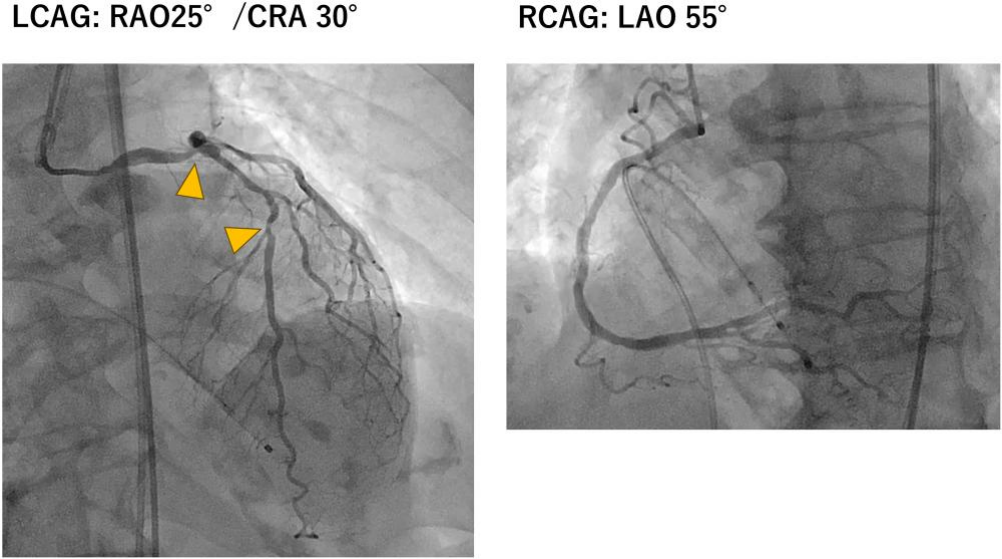
Coronary angiography of the left and right coronary arteries. Left coronary angiography (right anterior oblique, 25°/cranial, 25°) and right coronary angiography (left anterior oblique, 50°) reveal ∼50% stenosis in the left main coronary artery and 75% stenosis in the mid-left anterior descending artery. No remarkable stenosis is observed in the right coronary artery. An intracoronary injection of 1 mL nitroglycerine was administered prior to angiography. The sites of the left main coronary artery and left anterior descending artery stenoses are indicated by triangles.

**Figure 2:**
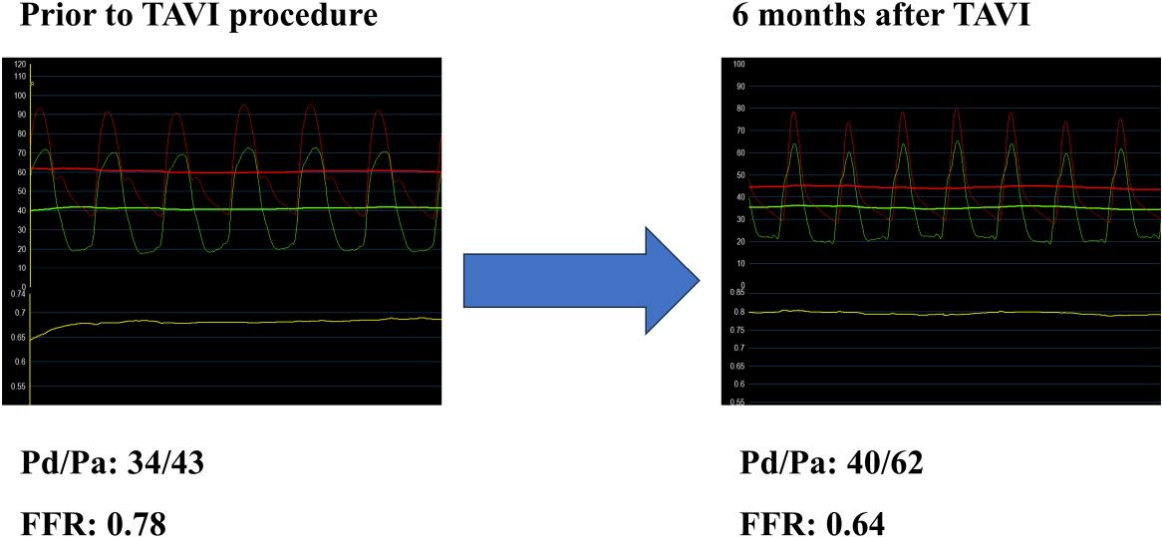
Changes in fractional flow reserve before and after transcatheter aortic valve implantation. Fractional flow reserve measurements performed with hyperaemia induced by intracoronary nicorandil in the left anterior descending artery before and 6 months after transcatheter aortic valve implantation. Pre-transcatheter aortic valve implantation: the fractional flow reserve is borderline at 0.78, suggesting functional equivocal ischaemia. Post-transcatheter aortic valve implantation: the fractional flow reserve has decreased to 0.64, indicating haemodynamically significant ischaemia unmasked by improved microvascular function following repair of aortic stenosis.

**Figure 3:**
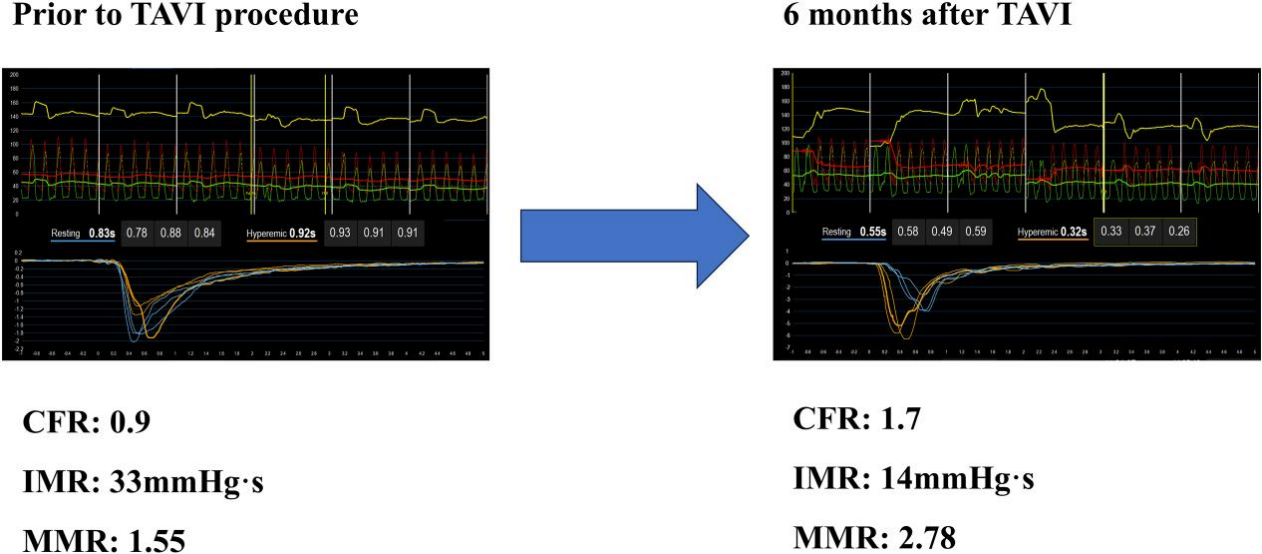
Coronary physiological assessment before and after transcatheter aortic valve implantation. Invasive coronary physiological measurements before and 6 months after transcatheter aortic valve implantation with hyperaemia induced by intracoronary nicorandil. Pre-transcatheter aortic valve implantation: the coronary flow reserve is 0.9, the index of microcirculatory resistance is elevated at 33, and the microvascular resistance reserve is 1.55, indicating significant coronary microvascular dysfunction. Post-transcatheter aortic valve implantation: the coronary flow reserve has improved to 1.7, the index of microcirculatory resistance has decreased to 14, and the microvascular resistance reserve has increased to 2.78, consistent with the recovery of microvascular vasodilatory capacity following aortic valve replacement.

Coronary angiography revealed no significant stenosis in the right coronary artery, 50% stenosis in the left main coronary artery (LMCA), and 75% stenosis in the proximal LAD (segment 7) (*Figure 1*). The FFR of the LAD, measured using intracoronary nicorandil as the hyperaemic agent, was 0.78 (*Figure 2*). Microvascular assessment demonstrated a coronary flow reserve (CFR) of 0.9, an IMR of 33, and a microvascular resistance reserve (MRR) of 1.55, consistent with significant coronary microvascular dysfunction (CMD) (*Figure 3*). The resting full-cycle ratio (RFR) was markedly reduced to 0.58, indicating a significant resting pressure gradient; however, the FFR was borderline at 0.78, and the IMR was elevated at 33. Given these findings, PCI was deferred.

### Procedural and post-procedural course

TAVI was performed under sedation using a 23 mm self-expanding NAVITOR valve (Abbott Structural Heart, Santa Clara, CA, USA) without peri-procedural complications. Post-procedural echocardiography showed marked haemodynamic improvement, with a peak velocity of 1.5 m/s, a mean pressure gradient of 5 mmHg, an effective orifice area of 2.2 cm², and only trivial aortic regurgitation.

**Figure 4:**
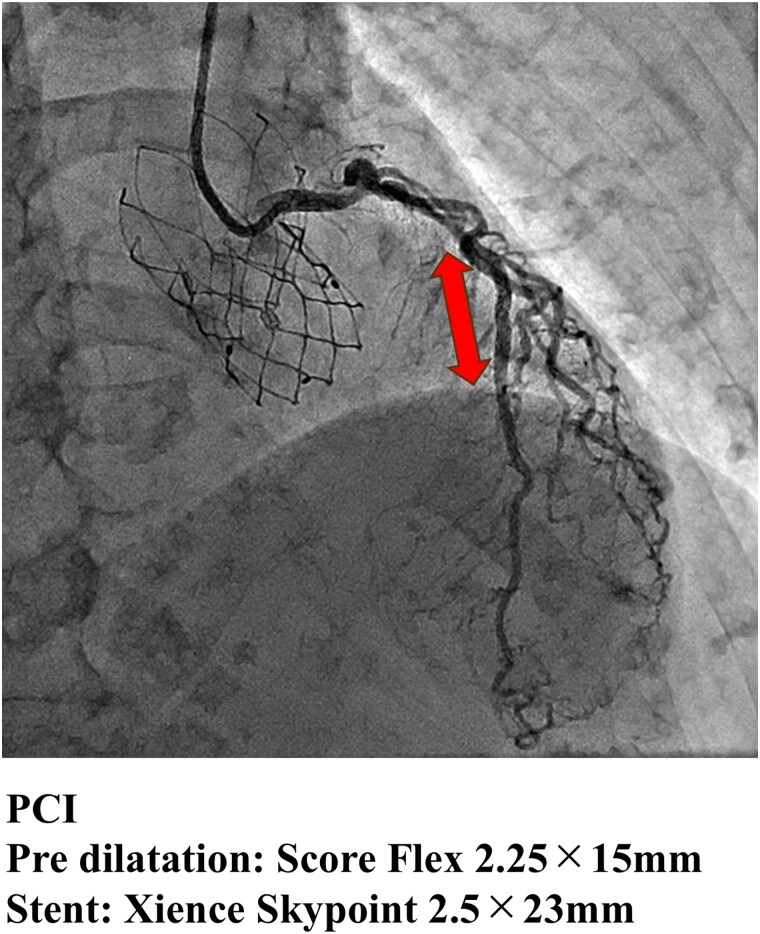
Percutaneous coronary intervention procedure. Percutaneous coronary intervention of the left anterior descending artery performed following the identification of fractional flow reserve-positive ischaemia. The lesion was prepared with a 2.25 × 15 mm cutting balloon (Scoreflex), followed by deployment of a 2.5 × 23 mm everolimus-eluting stent (Xience Skypoint). Final angiographic results show optimal stent expansion and TIMI 3 flow.

Following discharge, the patient reported partial improvement in symptoms (NYHA class II) but continued to experience exertional dyspnoea. Given the persistence of symptoms, a reassessment of her coronary status was planned at the 6-month follow-up visit. Repeat coronary angiography revealed no lesion progression, with LMCA and LAD stenoses remaining at 50% and 75%, respectively. However, the FFR value of the LAD had decreased to 0.64, while microvascular function had improved: CFR was 1.7; IMR, 14; and MRR, 2.78 (*Figure 3*). Based on these findings, PCI was performed using predilation with a 2.25 × 15 mm cutting balloon (Scoreflex), followed by implantation of a 2.5 × 23 mm everolimus-eluting stent (Xience Skypoint) (*Figure 4*).

**Figure 5:**
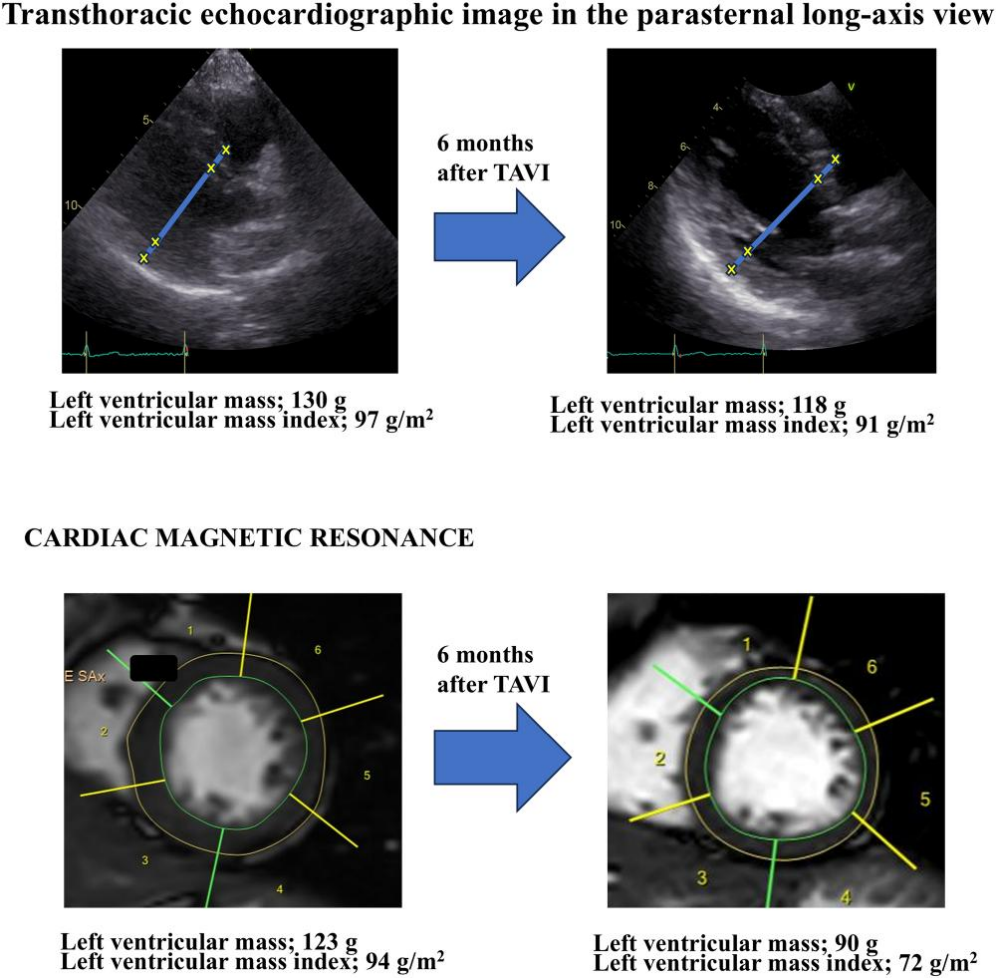
Left ventricular reverse remodelling assessed by echocardiography and cardiac magnetic resonance imaging. Structural changes in the left ventricle before and after transcatheter aortic valve implantation were assessed using transthoracic echocardiography and cardiac magnetic resonance imaging. Echocardiography: the left ventricular mass has decreased from 130 to 118 g, and the left ventricular mass index has decreased from 97 to 91 g/m². The interventricular septal thickness has also decreased from 11 to 8 mm. Cardiac magnetic resonance imaging: the left ventricular mass has decreased from 123 to 90 g, and the left ventricular mass index has decreased from 94 to 72 g/m². These findings indicate significant left ventricular reverse remodelling following relief from pressure overload after transcatheter aortic valve implantation.

Following PCI, her exertional dyspnoea resolved completely. Left ventricular reverse remodelling was documented using TTE and cardiac magnetic resonance imaging (MRI), as illustrated in *Figure 5*. These results are consistent with favourable reverse remodelling following TAVI, characterized by regression of myocardial hypertrophy and improved ventricular geometry.

## Discussion

The haemodynamic interplay between severe AS and CAD presents a diagnostic challenge, particularly in the assessment of intermediate lesions. In this case, the pre-TAVI physiological evaluation showed borderline FFR and elevated IMR, suggesting the presence of CMD.^4^ After TAVI, the FFR declined from 0.78 to 0.64, and the IMR improved from 33 to 14, indicating physiologically significant ischaemia and prompting PCI.^5^ In addition to FFR, the RFR was measured to assess non-hyperaemic physiology. The RFR showed a slight increase from 0.58 before TAVI to 0.62 after TAVI, whereas the FFR declined markedly from 0.78 to 0.64. This discrepancy may be explained by the recovery of hyperaemic microvascular function after TAVI, which enhances flow during maximal vasodilation but has a lesser impact on resting pressure gradients. Sabbah *et al*.^6^ previously reported that instantaneous wave-free ratio or RFR tends to change more than FFR after TAVI; however, our case demonstrated the opposite trend, likely reflecting an improvement in coronary microvascular reserve that primarily affects hyperaemic rather than resting flow. These findings suggest that post-TAVI physiological reassessment should include both hyperaemic and non-hyperaemic indices to comprehensively characterize coronary haemodynamic changes. AS increases afterload, leading to left ventricular hypertrophy and impaired microvascular vasodilatory capacity.^7^ This functional CMD may blunt hyperaemic flow and cause an underestimation of ischaemia by the FFR.^8^ TAVI alleviates afterload and reduces myocardial oxygen demand, promoting reverse remodelling and the restoration of coronary microvascular function.^9^ In this case, post-TAVI cardiac MRI confirmed regression of the left ventricular mass, consistent with previous studies showing a ∼20% reduction in the left ventricular mass index (LVMI) within 6 months of valve replacement.^9^

The observed improvements in the CFR and MRR further supported the functional recovery of the microvasculature.^10^ Before TAVI, the reduced CFR reflected the combined effects of structural CMD—driven by left ventricular hypertrophy and elevated filling pressures—and the presence of intermediate epicardial stenosis. After TAVI, the structural component of CMD improved, as evidenced by the normalization of IMR, and the low CFR mainly represented the residual influence of the epicardial lesion. Thus, while pre-TAVI CFR integrated both microvascular and epicardial contributions, post-TAVI CFR primarily reflected epicardial flow limitation. This physiological evolution indicates that TAVI can unmask the true functional significance of epicardial stenosis previously masked by CMD. As CMD resolved, the hyperaemic response normalized, and the true significance of the LAD lesion became apparent.^10^ In the present case, both CFR and IMR improved after TAVI, whereas Sabbah *et al*.^6^ reported that CFR increased significantly at follow-up while microvascular resistance remained unchanged. This discrepancy may be explained by differences in patient characteristics and the degree of CMD. In Sabbah *et al*.’s cohort, microvascular remodelling was likely structural, and the reduction in left ventricular afterload mainly improved CFR by lowering resting flow, without changing microvascular resistance. In contrast, our patient likely exhibited functional CMD, related to elevated filling pressure and extravascular compression. Thus, TAVI-induced afterload reduction and regression of left ventricular hypertrophy could have contributed to both a fall in IMR and an increase in CFR. These findings support the concept that CMD in severe AS is at least partly reversible following valve intervention. From a physiological perspective, multiple mechanisms may underlie the improvement in coronary microcirculation after TAVI. The marked reduction in left ventricular afterload decreases intramyocardial compressive forces and wall stress, thereby improving subendocardial perfusion. Restoration of a normal aortic pressure waveform and prolongation of diastolic perfusion time enhance coronary driving pressure, particularly in the microvasculature. Furthermore, regression of left ventricular hypertrophy reduces extravascular resistance and oxygen demand, facilitating more efficient flow distribution across the microcirculatory network. These changes can collectively lower microvascular resistance (reflected in IMR) and augment flow reserve (reflected in CFR).

In terms of calculation principles, CFR represents the ratio between hyperaemic and resting flow, while IMR quantifies the absolute resistance of microcirculation during hyperaemia.

MRR, as an integrated index derived from CFR and IMR, isolates microvascular vasodilatory capacity by accounting for epicardial resistance and aortic pressure effects.

Therefore, post-TAVI changes in these indices may not occur uniformly: CFR can rise due to increased hyperaemic flow or decreased resting flow, IMR decreases with true reduction in microvascular resistance, and MRR captures the net gain in microvascular reserve independent of epicardial conditions. This integrated physiological response reflects the reversal of functional CMD driven by reduced afterload, improved myocardial relaxation, and restored coronary autoregulation.

Given that CFR is influenced by epicardial stenosis and aortic pressure, MRR provides a more microcirculation-specific assessment of vasodilatory reserve. Recent evidence from Scarsini *et al*.^11^ demonstrated that in patients with severe AS, baseline MRR was significantly impaired—particularly in those with LFLG physiology—and that MRR markedly improved after TAVI, reflecting true recovery of microvascular function independent of epicardial resistance. This finding supports our observation that post-TAVI improvement in MRR indicates reversible microvascular dysfunction rather than simple changes in epicardial haemodynamics. Accordingly, we regard MRR as complementary rather than an alternative to CFR or IMR. While CFR reflects global vasodilatory capacity and IMR quantifies absolute microvascular resistance, MRR integrates both parameters to allow a more specific interpretation of microvascular function. Incorporating MRR into coronary physiological assessment before and after TAVI may enhance understanding of microvascular adaptation and refine revascularization strategies in patients with coexisting AS and CAD.

Observational studies have shown that residual ischaemia after TAVI is associated with adverse outcomes.^11^ For example, Scarsini *et al*.^11^ reported that post-TAVI CMD (IMR of ≥30) was linked to a six-fold increase in cardiovascular events. Therefore, a deferred PCI strategy with planned reassessment post-TAVI may be reasonable in patients with equivocal findings and suspected CMD. However, epicardial revascularization itself does not directly modify microvascular dysfunction. After correction of valvular afterload, persistent ischaemia with normalized IMR but low CFR likely reflects residual epicardial disease. In such cases, lesion-directed PCI may reduce ischaemia-related adverse events. Therefore, revascularization should be considered selectively, guided by comprehensive physiological reassessment rather than assumed to uniformly improve CMD. In conclusion, this case illustrated that CMD in patients with severe AS may be both functional and reversible. Incorporating CMD indices into pre-TAVI evaluations and considering reassessment in selected patients may help guide optimal revascularization strategies and improve outcomes.

This case involved an elderly patient with paradoxical LFLG severe AS and intermediate LAD stenosis, in whom CMD was identified prior to TAVI. Borderline FFR and elevated IMR led to deferred revascularization. Following TAVI, improvements in microvascular function unmasked physiologically significant ischaemia, prompting PCI and symptom resolution. This case highlights that in severe AS, coronary physiological assessment prior to TAVI may underestimate ischaemia due to CMD. Post-TAVI reassessment is essential in selected patients, particularly when the FFR is borderline and the IMR is elevated. Improvements in the microvascular function after TAVI can reveal haemodynamically significant lesions and guide appropriate revascularization, ultimately leading to symptom relief and improved outcomes.

## Supplementary Material

ytaf649_Supplementary_Data

## Data Availability

All data relevant to the case are included in the article.

## Lead author biography

Dr Kosuke Fujita graduated from Kindai University Faculty of Medicine in 2010 and completed his residency at Kindai University Hospital. He joined the Department of Cardiology in 2012 and obtained his PhD in 2017. From 2018 to 2020, he studied abroad at the University Magna Graecia of Catanzaro in Italy. He currently serves as a cardiologist at Kindai University Hospital, specializing in ischaemic heart disease, catheter-based interventions, and structural heart disease, including TAVI.

## Supplementary material

**Consent:** Written informed consent was obtained from the patient for publication of this case report and accompanying images, in compliance with Committee on Publication Ethics (COPE) guidelines.
