# Supplementary material for: Improvement in coronary microvascular dysfunction after transcatheter aortic valve implantation leading to positive fractional flow reserve and percutaneous coronary intervention: a case report
Source: Eur Heart J Case Rep. 2025 Dec 13;10(1):ytaf649. doi: 10.1093/ehjcr/ytaf649 (PMC12780881; doi:10.1093/ehjcr/ytaf649)
Supplement: ytaf649_Supplementary_Data [file ytaf649_supplementary_data.docx]

# Supplementary Table 1. Echocardiographic parameters before TAVI

| Parameter | Value | Unit / Description |
| --- | --- | --- |
| LVDd / LVDs | 39 / 26 | mm |
| IVS / LVPW | 10 / 11 | mm |
| LVMi | 97 | g/m² |
| LVEF | 66% | No apparent wall motion abnormality |
| SVi | 42 | mL/m² |
| AV Vmax | 3.5 | m/s |
| Mean PG | 30 | mmHg |
| AVA (continuity equation) | 0.68 | cm² |
| MR | Trivial |  |
| AR | Mild |  |
| TR | None |  |

Abbreviations: LVDd = left ventricular diastolic diameter; LVDs = left ventricular systolic diameter; IVS = interventricular septum; LVPW = left ventricular posterior wall; LVMi = left ventricular mass index; LVEF = left ventricular ejection fraction; SVi = stroke volume index; AV = aortic valve; PG = pressure gradient; AVA = aortic valve area; MR = mitral regurgitation; AR = aortic regurgitation; TR = tricuspid regurgitation.

**Supplementary Table 1.** Echocardiographic parameters before transcatheter aortic valve implantation (TAVI).
The table summarizes pre-procedural echocardiographic findings, including left ventricular dimensions, wall thickness, systolic function, stroke volume index, and valvular hemodynamics. No regional wall motion abnormalities were observed, and mild aortic regurgitation with trivial mitral regurgitation was noted.
